# Ethnobotanical Study of Mosquito Repellent Plants Used in Seweyna District, Bale Zone, Southeast, Ethiopia

**DOI:** 10.1155/2024/6610579

**Published:** 2024-03-15

**Authors:** Asefash Shibeshi, Ayalew Sebsibe, Alemtshay Teka, Esayas Aklilu

**Affiliations:** ^1^Mada Walabu University, Bale Robe, Ethiopia; ^2^Aklilu Lemma Institute of Pathobiology, Addis Ababa University, Addis Ababa, Ethiopia

## Abstract

Malaria control efforts through vector control strategies are hindered by the development of insecticide resistance by major malaria vectors in many malaria-endemic areas, which necessitate the need for alternative control measures. The aim of this study was to document plants traditionally used as mosquito repellents in Seweyna district, southeastern Ethiopia. The ethnobotanical data were collected using semistructured interviews, field observation, and guided field walks in four kebeles of the district with 98 informants. A total of 19 plant species were used by the local community as mosquito repellent, with 42.1% being trees. These plant species belong to 12 families. Of these families, the family Burseraceae was the most represented, with four species, followed by Fabaceae (3 species). The most frequently mentioned plant species were *Mimusops kummel* (90.81%), followed by *Acokanthera schimperi* (84.69%), *Boswellia microphylla* (79.6%), and *Calpurnia aurea* (79.6%). The stem was the most common plant part used (47.3%) to repel mosquitoes. Most of the local communities (52.6%) use the burning of either fresh or dry plant parts to generate smoke, which is the most common practice. The current ethnobotanical study indicates that the local community in the Seweyna district uses the plants to repel mosquitoes. In the future, the repellent efficacy of these plants against the major malaria vector should be tested under laboratory and field conditions. Besides, the identification of the bioactive compounds responsible for the repellent activity should also be determined.

## 1. Introduction

Mosquito-borne diseases, such as dengue, yellow fever, zika, chikungunya, malaria, and others, are the major sources of morbidity and mortality for several millions of people. Malaria is a major public health problem in tropical and subtropical countries of the world with an estimated 247 million clinical cases and 619 000 deaths in 2021. Ninety-two and ninety-three percent of the cases and deaths, respectively, occurred in Sub-Saharan African (SSA) countries [1].

In Ethiopia, as in other SSA countries, malaria is one of the top public health problems with millions of cases and thousands of fatalities reported each year [2]. The country has a population of more than 110 million, and it is estimated that approximately 52% of the population is at risk of the disease [3]. *Anopheles arabiensis*, a member of the *An. gambiae* species complex, is the principal vector whereas *An. pharoensis*, *An. funestus*, and *An. nili* are the secondary vectors of the disease in the country [4].

Vector control, achieved through indoor residual spraying (IRS) and large-scale distribution of long-lasting insecticidal nets (LLINs), is one of the currently implemented malaria control strategies in Ethiopia. This method, along with other malaria control strategies, has reduced malaria-related illness and mortality in the country [2, 5]. Despite reports stating the efficacy of the vector control methods (IRS and LLINs), insecticide resistance in malaria vectors poses a serious threat to the success of malaria vector control programs in SSA [6–10]. In addition, various studies have pointed out that IRS and LLINS have a limited effect on malaria vectors that are active during the early hours of the night and have an exophagic feeding behavior [11–14]. Furthermore, the chemical insecticides used to prepare the IRS and LLINs may pose a serious threat to the health of humans and the environment [15]. Consequently, to overcome the aforementioned limitations, there is a need to develop new vector control methods that complement the currently used vector control approaches.

Insect repellents of plant origin have been used to protect humans from the bites of host-seeking mosquitoes [16]. Such repellents may have a fundamental role in areas where malaria vectors are active during the early hours of the night, as the inhabitants are often outdoors at these times. Thus, they may serve as a complementary method to the indoor-based vector control method [17].

The use of traditional repellents to repel/kill mosquitoes and other blood-sucking insects is common in rural communities in Africa [18]. For example, in Kenya, direct burning and placing branches or whole plants are the common methods to drive away mosquitoes [18]. In Eritrea, hanging the different plant parts on the walls at the head and foot of beds reduces endophagic mosquitoes [19]. In Tanzania, people burn or place fresh plant materials at selected places or spray to decrease the number of mosquitoes indoors at night [20]. In different parts of Ethiopia, smoking by burning various parts of repellent plants is the principal way of deterring nuisances and biting insects [17, 21].

Documentation of indigenous knowledge and the use of plants as repellents for mosquitoes/insects are very essential for different reasons. The first reason is that it is a precondition for future research on the development of a new repellent or an insecticidal compound [15]. The second reason is that such knowledge and practice are transferred from one generation to the next generation mostly through the word of mouth. However, this way of conveying indigenous knowledge and cultural practices may face distortion or extinction due to different factors [22]. Hence, this study was carried out to assess the knowledge and usage customs of traditional insect/mosquito repellent plants in Seweyna district, Bale Zone, Southeast Ethiopia, where malaria is endemic (Bale Zone Health Bureau unpublished data).

## 2. Materials and Methods

### 2.1. Description of the Study Area

**Figure 1:**
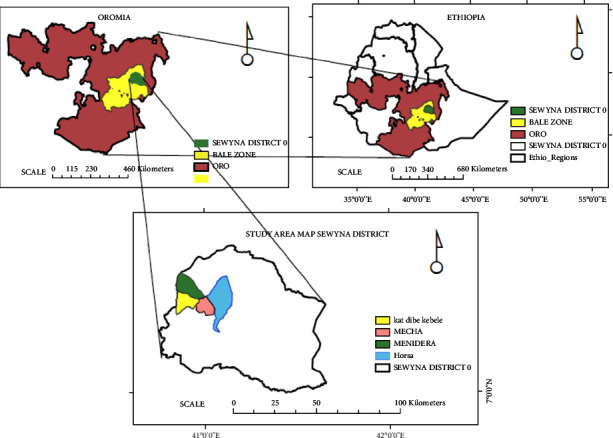
Map of the study area.

Seweyna district is located 7°19′60″ N and 41°19′60″ E of Bale Zone in Oromia Regional State, southeast Ethiopia (Figure 1). It is situated about 619 km away from Addis Ababa, at an altitude of approximately 1800 meters above sea level (masl) and the mean annual temperature and annual rainfall of the district are about 30^0^ C and 700 mm, respectively. The rainy seasons are from September to November and March to June. In the majority of the area, the vegetation type is bushy shrubs. According to the Central Statistical Agency report (2005), the total population of the district was estimated to reach 49,381. The majority of the population belongs to the Oromo ethnic group and follows the Muslim religion. Many of the people in the district depend on the raising of cattle as a means of subsistence. In addition, the people are producing different crops such as maize, sorghum *teff,* and *khat* (*Catha edulis*).

### 2.2. Study Site and Informant Selection

Ethnobotanical survey was conducted in Seweyna district from May to September 2018. The district has 19 kebeles (the smallest administrative unit). For the survey, four kebeles (Micha, Mandera, Katta Dibbe, and Horsa) were selected purposefully. The kebeles were selected based on the medicinal plant use practices of the community and the previously reported high number of malaria cases (Seweyna Health Center unpublished data). A total of ninety-eight informants (88 general and 10 key informants) were selected. The general informants who volunteered to participate were selected randomly by a lottery method. In order to collect additional specific quantitative data, key informants who were acknowledged by the local community elders, religious leaders, and local administrators were purposefully selected [23].

### 2.3. Ethnobotanical Data Collection

Ethnobotanical data were collected using semistructured interviews, field observation, and guided field walks with the informants. The questionnaire was prepared in English and translated into the common language, Afan Oromo. The informants were asked about the plants used to repel mosquitoes, the plant parts used, the mode of application, the condition of the plant parts used (dried or fresh), and the method of preparation.

### 2.4. Plant Collection and Identification

All reported plant specimens were collected, dried, and identified at the National Herbarium of Addis Ababa University. The plants were identified using the Flora of Ethiopia and Eritrea in comparison with authenticated specimens from the Herbarium and later confirmed by senior taxonomists of the Herbarium. The voucher specimens were deposited at Madda Walabu University, Ethiopia.

### 2.5. Ethical Consideration

Permission to conduct the study was obtained from the Seweyna district administration office and community leaders. The purpose of the study was explained to the informants prior to the interview and informed verbal consent was obtained from each informant.

### 2.6. Data Analysis

The collected ethnobotanical data were entered into SPSS version 20 and summarized using descriptive statistics. Descriptive statistical methods (percentage and frequency) were used to summarize the traditional knowledge of the community related to the plants used to repel mosquitoes, the plant parts used, the condition of the plant parts used, the method of preparation, and the mode of application. In addition, the local importance of each species was analyzed by the relative frequency of citation (RFC) which is expressed by the total number of informants mentioning the use of the species (the frequency of citation (FC)) divided by the total number of informants (*N*).

(1) $$\begin{array}{c} \text{RFC}=\frac{\text{FC}}{N}*100. \end{array}$$

Furthermore, a preference ranking of seven medicinal plants that were indicated as effective mosquito repellents in the study area was conducted using 10 key informants. All informants were asked to compare the plants based on their efficacy and to mark the highest value (7) for the most preferred and the lowest value (1) for the least preferred.

## 3. Results

### 3.1. Sociodemographic Characteristics of the Informants

The sociodemographic characteristics of the study informants are presented in Table 1. Of the total informants, 92.9% were male and 7.1% were female. The age of the informants ranged from 31 to 81 years with the majority between 41 and 60 years old. 80.61% of the informants were illiterate and 42.9% of them had a monthly income between 201 and 400 Ethiopian Birr. 88% of the informants were farmers and all of the informants belonged to the Muslim religion (Table 1).

### 3.2. Diversity and Source of Plant Species Used for Repelling Mosquito

A total of 19 plant species were used by the local community as a mosquito repellent in the district (Table 2). These plant species belong to 12 families. Of these families, the family Burseraceae was the most represented family with four species followed by Fabaceae (3 species). The family Combretaceae and Solanaceae were represented by two species each, while the remaining families consisted of a single species each. The majority of plant species (42.1%) used as mosquito repellent were trees, while 31.58% and 26.32% were shrubs and herbs, respectively.

**Figure 2:**
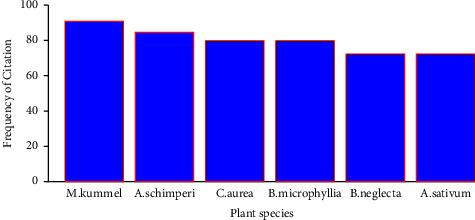
Most frequently cited plants used as a mosquito repellant in Seweyna district.

The most frequently mentioned plant species were *Mimusops kummel* mentioned by 89 (90.81%) and *Acokanthera schimperi* mentioned by 83 (84.69%) informants. *Calpurnia aurea* and *Boswellia microphylla* were cited by 72 (79.6%) informants each and *Boswellia neglecta* and *Allium sativum* were mentioned by 71 (72.4%) informants each (Figure 2).

### 3.3. Plant Parts Used, State of Plant Material, and Method of Application of Mosquito Repellent Plants

**Figure 3:**
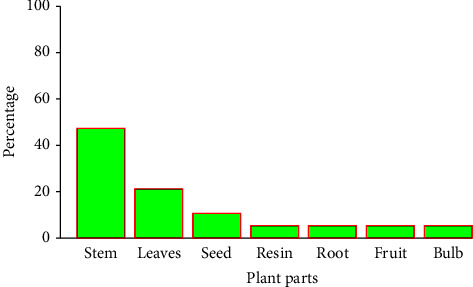
Plant parts used for repelling mosquitoes.

**Figure 4:**
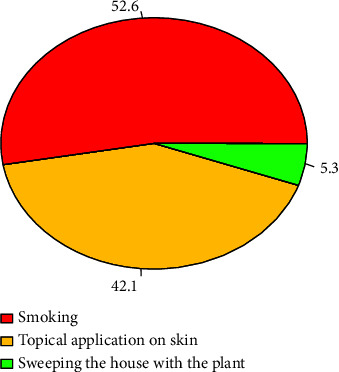
Method of application to repel mosquito.

The stem was the most common plant part used (47.3%) to repel mosquitoes followed by the leaves (21%) and seed (10.5%) (Figure 3). The majority of plant parts (80%) were used in dried form. Most of the local communities (52.6%) used burning of either fresh or dry plant parts to generate smoke as the most common practice, followed by topical application on the skin (42.1%) and by sweeping the house with the plant (5.3%) (Figure 4).

### 3.4. Preference Ranking of Medicinal Plants Used as Mosquito Repellent

The preference ranking of the seven frequently cited plants used for repelling mosquitoes in the study area showed that *Acokanthera schimperi* was the most preferred by the local people followed by *Mimusops kummel* (Table 3).

## 4. Discussion

This study attempted to document knowledge about plant species used as repellents against mosquitoes in Seweyna district, southeastern Ethiopia where malaria is endemic, to identify the potential candidate that might be formulated as insect repellent. In the present study, a total of 19 plant species were reported to be used as mosquito repellents in the study area. As compared to the previous studies conducted in various parts of the country [17, 24, 25], a relatively higher number of insect-repellent plants were recorded in the present study district. This signifies that the area consisted of a considerable diversity of plant species. Of the total plant species recorded, the families Burseraceae and Fabaceae contributed four and three species, respectively. These plant families were previously reported in different parts of Ethiopia [17, 21, 25–27]. This might indicate that these families have a wider geographical distribution and rich bioactive substances that play a significant role in the repellent activity of the plant.

The most repeatedly cited mosquito-repellent plant was *Mimusops kummel* (90.81%). The use of this plant as an insect repellent and/or insecticidal has also been reported in the Raya-Azebo district, northern Ethiopia [28]. Such high agreement among the study participants and similar reports with communities in different areas may signify that the plant species has a better efficacy and appears to contain more biologically active ingredients as compared to plants with less frequency of citation in the present study, although no previous study has analyzed the phytochemical effect of this plant against mosquitoes.

*Acokanthera schimperi*, which is native to Yemen and East Africa, is the second repeatedly cited and most preferred mosquito repellent plant by the local community in the study area. Such plants have been used for similar purposes by pastoral and agropastoral communities in Dengego, Erer, and Harla Valleys in Eastern Ethiopia [29, 30]. In addition, the *in vivo* antimalarial activity of the leaf extract of *Acokanthera schimperi* against *Plasmodium berghei* was evaluated in Ethiopia and showed significant suppression of the parasitaemia [31]. Furthermore, in Kenya, the larvicidal activity of the plant species was tested against the larvae of two tick species (*Rhipicephalus appendiculatus* and *Boophilus decoloratus*) and resulted in a moderately high mortality in the larvae of the tick species [32].

*Calpurnia aurea* is a member of the family Fabaceae and is widespread in sub-Saharan Africa and India [33]. In the current study, the plant is the third most preferred mosquito repellent. In accordance with the present study, the community in Jabi Tehnan district, northwestern Ethiopia, used *C. aurea* as one of the mosquito repellent plants [26]. Likewise, people in Kolla Temben district, northern Ethiopia, used the plant for a similar purpose. Besides documenting its mosquito-repellent effect, studies have been conducted to evaluate its larvicidal effect under laboratory conditions. Eukubay et al. [34] evaluated the methanol leaf extract of *C. aurea* at concentrations ranging from 50 to 300 ppm against 3^rd^ instar *An. arabiensis* larvae and found 100% mortality at the maximum concentration. Similarly, Muhammed et al. [35] assessed the larvicidal effects of aqueous, hexane, and methanol crude leaf extracts of *C. aurea* with various concentrations ranging from 25 ppm to 300 ppm against the third to fourth instar larvae of *An. stephensi*. The authors reported that 100% mortality was found in aqueous and methanol extracts with high concentrations (200–300 ppm). Such a high biological activity might be attributed to the presence of bioactive secondary metabolites, including phenolic, tannin, alkaloids, flavonoids, and saponin [36].

The results of the current study with respect to the parts of the plants used for driving away the mosquitoes indicated that the local communities mainly used the stems (47.3%), followed by leaves (21%). This result is in agreement with the previous reports [26, 29]. Such repeated use of stem parts in the study area can threaten the survival of local medicinal plants unless a sustainable harvest mechanism is developed [37].

The most favored method of application to repel mosquitoes was burning the plant parts and smoking in the house. Similar findings have been reported in the previous studies in Ethiopia [17, 29], Eritrea [19], Kenya [18], Guinea Bissau [38], and Burundi [39]. Despite the fact that there is no detailed study which indicates how repellent smokes or their constituents act, Dube et al. [40] have proposed the following possible mechanisms for the action of the plant derived on the insects. The smoke may conceal the chemical substances that come out of the human body, which is important for the mosquitoes to locate their hosts, and it may interrupt the convection currents vital for mosquitoes to host location; and burning the plant parts may release volatile compounds, either already present in the plant parts or created during the burning process, that act as repellents or irritants against mosquitoes.

## 5. Conclusions

The current ethnobotanical study indicates that the local community in the Seweyna district uses the plants to repel mosquitoes. A total of nineteen plant species were reported. Of these, *Acokanthera schimperi*, *Mimusops kummel,* and *Calpurnia aurea* were the most preferred plant species used as mosquito repellent in the district. In the future, the repellent efficacy of these preferred plants against the major malaria vector should be tested under laboratory and field conditions. Besides, the identification of the bioactive compounds responsible for the repellent activity should also be determined.

## Figures and Tables

**Table 1:** Sociodemographic characteristics of participants.

Variable	No. of informants	Percentage
*Gender*		
Male	91	92.9
Female	7	7.1
*Age*		
31–50	26	26.5
51–60	40	40.8
>60	32	32.7
*Educational status*		
Illiterate	79	80.61
Literate	19	19.39
*Occupation*		
Farmer	88	88
Merchant	9	9
Civil servant	1	1
*Income*		
<400	78	79.8
400–800	18	18
>800	2	2

**Table 2:** List of medicinal plants used as mosquito repellants in Seweyna district.

No	PF	SN	VN	FC	FC in %	GF	PU	CU	MOP and MOA
1	Alliaceae	*Allium sativum* L	Qullubi adii	75	76.53	H	Bulb	Dried	Crushing to apply on the dermal
2	Apocynaceae	*Acokanthera schimperi* (A.DC.) Schweinf	*Qaraaru*	83	84.69	S	Leaves	Fresh	Burning and macerating in water
3	Brassicaceae	*Lepidium sativum* L	*Feexoo*	48	48.98	H	Seed	Dried	Smash to apply on the dermal
4	Burseraceae	*Commiphora myrrha* Engl	Qumbii	69	70.4	T/S	Resin	Dried	Burning the resin
5	Burseraceae	*Boswellia microphylla* Chiov	Dakkara	72	73.47	T	Stem	Dried	Burning to generate smoke
6	Burseraceae	*Boswellia neglecta S.* Moore	*Mugloo*	71	72.45	T	Stem	Dried	Burning to generate smoke
7	Burseraceae	*Commiphora guidottii* Chiov	Unsii	61	62.24	S	Stem	Dried	Burning dry stem to smoke
8	Combretaceae	*Terminalia* sp	Gabroo	68	69.39	T	Stem	Dried	Burning dry stem to smoke
9	Combretaceae	*Terminalia brownii* Fresen	Birreessa	70	71.43	T	Stem	Dried	Burning dry stem to smoke
10	Ebenaceae	*Euclea racemosa* Murray	Mi'eesa	60	61.22	S	Leaves	Fresh	Burning fresh leave
11	Fabaceae	*Calpurnia aurea* (ait.) Benth	*Ceekata*	72	73.47	S	Leaves	Fresh	Hang and sweep with it
12	Fabaceae	A*cacia bussei* Harms.ex.Joste	Hallo	18	18.37	T	Stem	Dried	Making oil from the stem to apply on the dermal
13	Fabaceae	*Acacia mellifera* Benth	Bilaala	18	18.37	S	Stem	Dried	Making oil from the stem to apply on the dermal
14	Lamiaceae	*Ocimum ellenbeckii* (Gurke)	Urgoo	56	57.14	H	Leaves	Dried	Grinding mix with butter to apply on the dermal
15	Rutaceae	*Citrus aurantifolia* (Christm)	Loomii	63	64.29	T	Fruit	Fresh	Crushing to apply on the dermal
16	Sapotaceae	*Mimusops kummel* Bruce ex A.DC	Qolaati	89	90.81	T	Stem	Dried	Burning to generate smoke
17	Simaroubaceae	*Kirkia* sp	Bisdhuga	51	52.04	T	Stem	Dried	Burning dry stem to apply on the dermal
18	Solanaceae	*Solanum* sp	Hiddi re'e	56	57.14	H	Seed	Dried	Grinding mix with water to apply on the dermal
19	Solanaceae	*Withania somnifera* (L.) Dunal	Hunjoo	67	68.37	H	Root	Dried	Grinding mix with butter to apply on the dermal

PF, plant family; SN, scientific name; VN, vernacular name; FC, frequency of citation; GF, growth form; PU, plant parts used; CU, condition of use; methods of preparation; GF, growth form; T, tree; S, shrub; H, herb; F, fresh; D, dried.

**Table 3:** Preference ranking of plants used as mosquito repellent.

Plants species	Key informants labeled A–J										Total score	Rank
	A	B	C	D	E	F	G	H	I	J		
*Acokanthera schimperi*	7	6	7	7	7	7	6	7	6	7	67	1^st^
*Mimusops kummel*	6	4	6	6	6	6	7	6	7	6	60	2^nd^
*Calpurnia aurea*	2	7	1	4	5	5	4	5	4	3	40	3^rd^
*Boswellia microphylla*	5	5	5	1	1	4	1	3	3	5	33	4^th^
*Commiphora myrrha*	4	1	4	5	2	2	2	1	5	4	30	5^th^
*Boswellia neglecta*	1	3	3	3	4	3	5	2	2	1	27	6^th^
*Terminalia brownii*	3	2	2	2	3	1	3	4	1	2	23	7^th^

## Data Availability

The data used to support the findings of this study are included within the article.
